# Supplementary figures and images for: Lamprey VLRB response to influenza virus supports universal rules of immunogenicity and antigenicity
Source: eLife. 2015 Aug 7;4:e07467. doi: 10.7554/eLife.07467 (PMC4552221; doi:10.7554/eLife.07467)

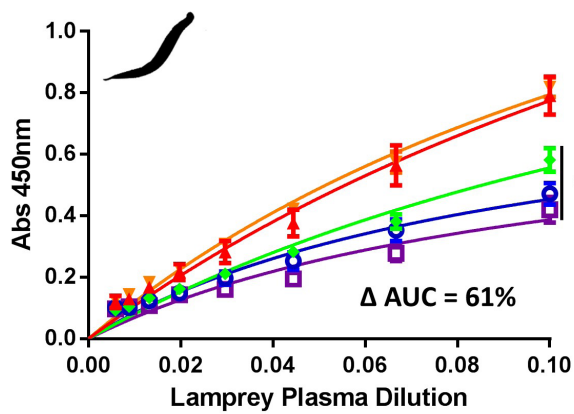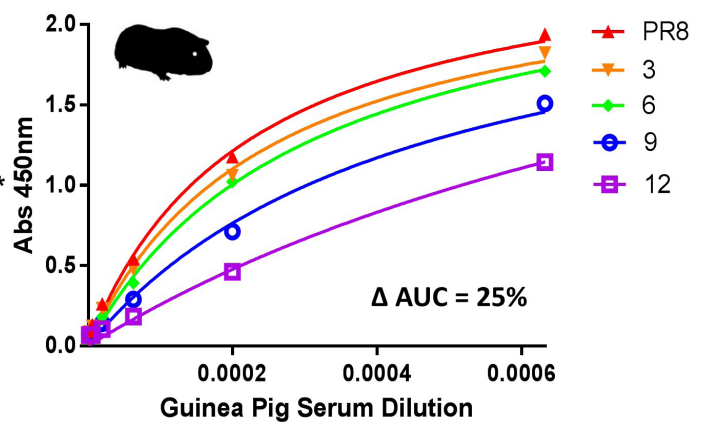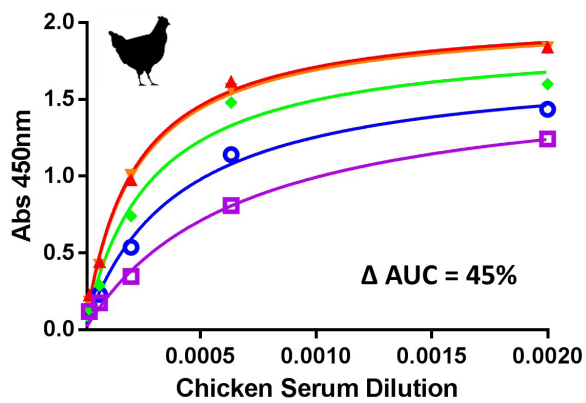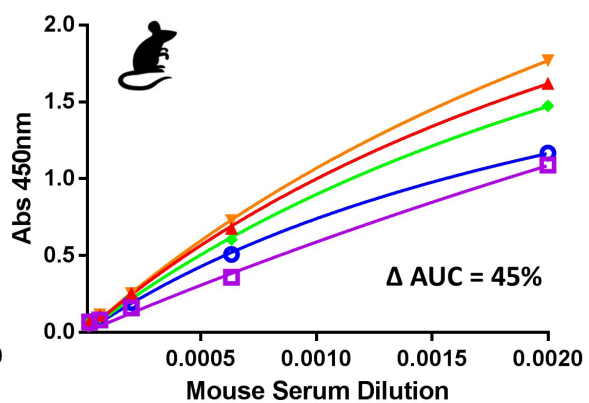

Supplement: Table 1—source data 1. — Other animals show similar binding to sequential virus series. ELISA binding curves for lamprey plasma from Table 1 are plotted alongside PR8 immunized guinea pig, chicken and mouse sera against the same plated Sequential virus series. Each graph shows representative data on a single animal's sera. Percent change in area under curve between wt PR8 and Sequential 12 is shown on each graph (ΔAUC). DOI: http://dx.doi.org/10.7554/eLife.07467.011 [file elife07467s001.pdf]

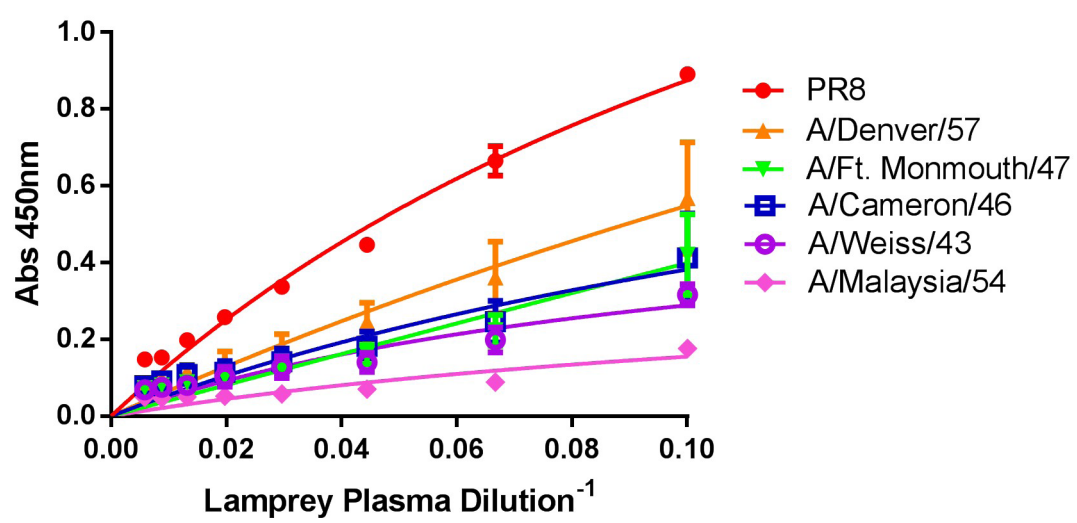

Supplement: Table 1—source data 2. — More H1N1 isolates. ELISA binding curves for lamprey plasma from Table 1 against plated H1N1 isolates are plotted along with two additional isolates omitted from the Table. DOI: http://dx.doi.org/10.7554/eLife.07467.012 [file elife07467s002.pdf]

**A**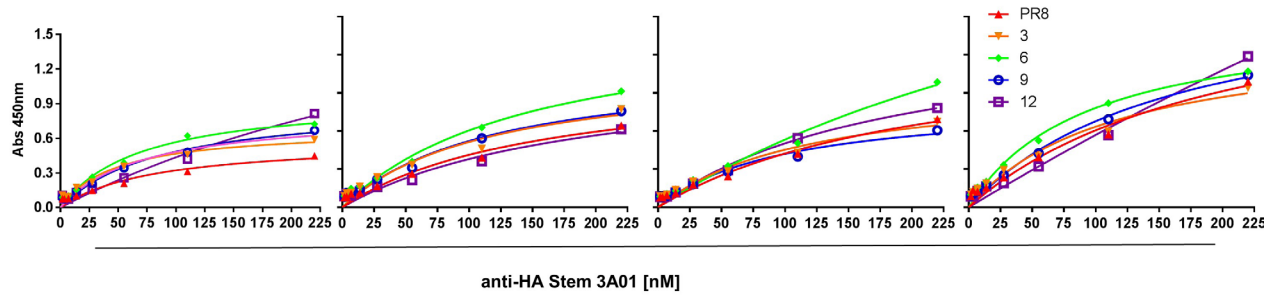**B**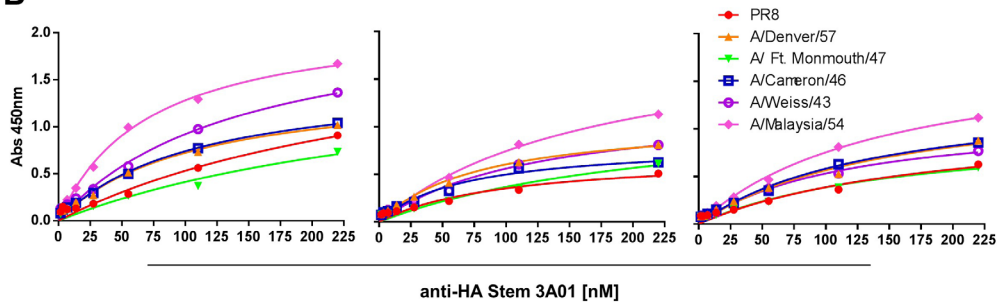

Supplement: Table 1—source data 3. — Anti-HA stem Ab binding curves used to normalize amounts of plated HA in Table 1. (A) ELISA binding curves for serially diluted anti-HA stem Ab (3A01) added to the Sequential virus series are show from each of the four experiments. (B) Same as (A) for the H1N1 isolate panel. DOI: http://dx.doi.org/10.7554/eLife.07467.013 [file elife07467s003.pdf]

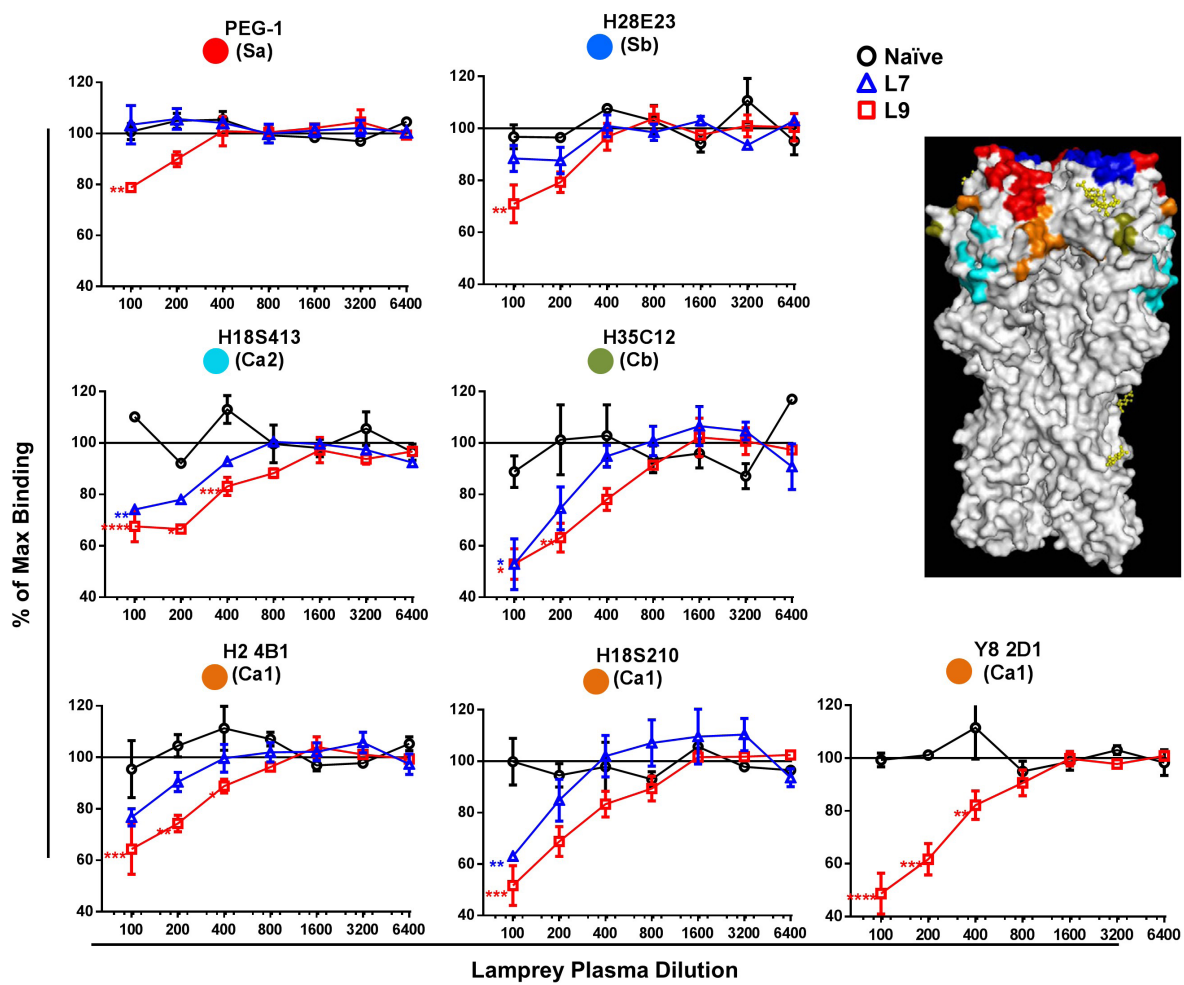

Supplement: Table 2—source data 1. — Competition ELISA against α-Head HA panel Abs. Data from Table 2 shown in graph form. Serially diluted unlabeled lamprey plasma raised against PR8 (L7, L9 or Naïve) was added to PR8 immobilized on 96 well ELISA plates. After 1 hr incubation, a fixed concentration of each indicated hybridoma supernatant (PEG-1, H28E23, H18 S413, H35 C12, H2 4B1, H18 S210, and Y8 2D1) was added at a predetermined concentration—65% of maximum binding (EC65). Data from three independent experiments were analyzed by Two Way ANOVA followed by Bonferroni Multiple Comparisons against the Naïve plasma data using PRISM. (*p < 0.05; **p < 0.01; ***p < 0.001; ****p < 0.0001). DOI: http://dx.doi.org/10.7554/eLife.07467.015 [file elife07467s004.pdf]

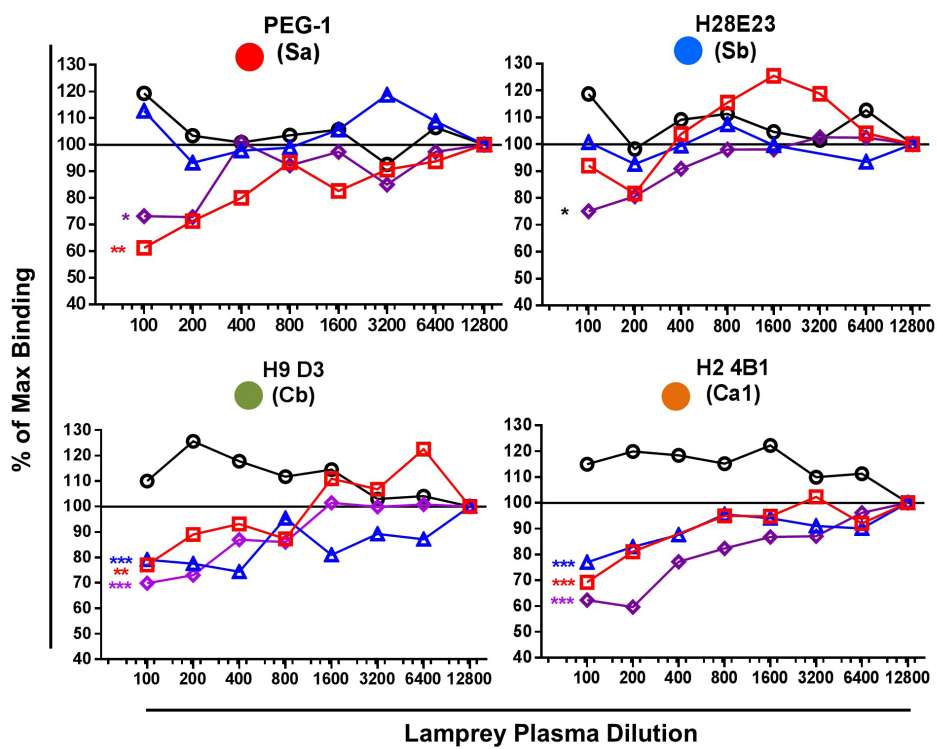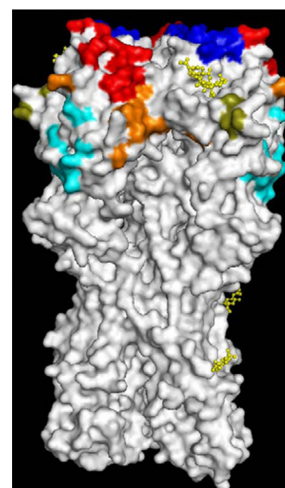

Supplement: Table 2—source data 2. — Competition ELISA against α-Head HA Fabs. Same as Table 2—source data 1 but with Fabs instead of hybridoma supernatants. p-value measurements determined with One-Way Anova followed by Dunnett's Multiple Comparison Test against Naïve plasma values. Stars indicate differences among whole groups. Data collected from only one experiment due to shortage of lamprey plasma. DOI: http://dx.doi.org/10.7554/eLife.07467.016 [file elife07467s005.pdf]

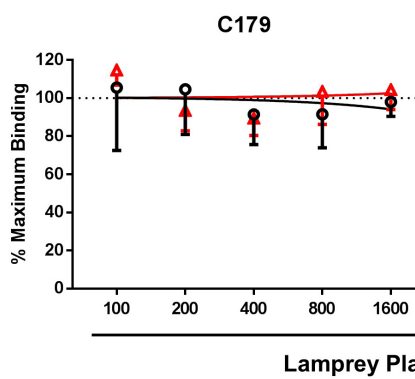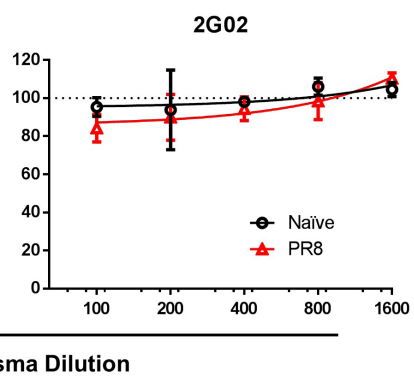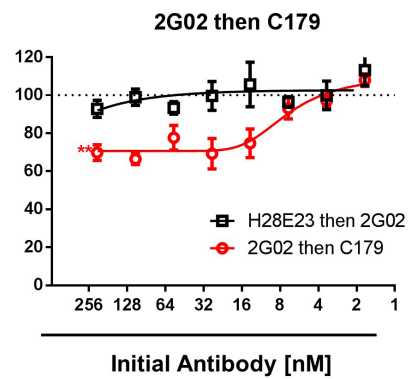

Supplement: Table 2—source data 3. — Immune lamprey plasma does not compete against stem binding Abs by ELISA. Serially diluted naïve or immune lamprey plasma raised against PR8 (L29) on 96 well ELISA plates immobilized with PR8. After 1 hr incubation, a fixed concentration of purified monoclonal C179 or 2G02 was added at EC65. As a positive control, the two stem Abs were competed against each other or against an anti-HA head Ab (H28E23). Data are from at least two separate experiments with four total replicates. There was no statistical difference between the lamprey plasma curves. ELISA signal from these Abs is low, thus the curves are noisy. In contrast, the ‘2G02 then C179 curve’ is statistically different from the ‘H28E23 then 2G02 curve’ by two-tailed t-test (**p< 0.01). DOI: http://dx.doi.org/10.7554/eLife.07467.017 [file elife07467s006.pdf]
